# Determination of Metals Content in Wine Samples by Inductively Coupled Plasma-Mass Spectrometry

**DOI:** 10.3390/molecules23112886

**Published:** 2018-11-05

**Authors:** Justyna Płotka-Wasylka, Marcin Frankowski, Vasil Simeonov, Żaneta Polkowska, Jacek Namieśnik

**Affiliations:** 1 Department of Analytical Chemistry, Faculty of Chemistry, Gdańsk University of Technology, 11/12 Narutowicza Street, 80-233 Gdańsk, Poland; zanpolko@pg.gda.pl (Ż.P.); chemanal@pg.edu.pl (J.N.); 2 Department of Water and Soil Analysis, Faculty of Chemistry, Adam Mickiewicz University in Poznań, Umultowska 89 b, 61-614 Poznań, Poland; marcin.frankowski@amu.edu.pl; 3 Faculty of Chemistry and Pharmacy, University of Sofia, 1 James Bourchier Blvd., 1126 Sofia, Bulgaria; vsimeonov@chem.uni-sofia.bg

**Keywords:** wine, metals, soil, chemometric analysis, ICP-MS, ICP-OES

## Abstract

Knowledge about the metal content of wine is very important, for many reasons. Depending on the element, its quantity varies in wine from ng/L to mg/L. Despite the fact that metals are not directly connected to the taste and aroma of the wine, their content should be determined and controlled, because excess is undesirable, and in some cases prohibited, due to potential toxicity. Several analytical procedures for metal determination are applied. However, due to sensitivity, low limit of detection and speed of analysis, inductively coupled plasma-mass spectrometry (ICP-MS) is one of the most frequently used techniques. The aim of this study was to reveal specific relationships between the wine samples or between the chemical variables in order to classify the wines according to their metal content by application of chemometric analysis. For metals content determination, two techniques, ICP-MS and inductively coupled plasma-optical emission spectrometry (ICP-OES), were applied. Data obtained showed that none of the wine samples surpassed the toxic levels reported for metals in the literature, thus, these wines appeared to be safe as regards the risk associated with the potentially toxic metals intake. However, specific correlations between metals and specific aspects of the wines themselves have been found.

## 1. Introduction

Without a doubt, determination of metals in different type of food samples is of high importance for several reasons, with the most important one being the nutritional, as well as toxic, effects of these elements or their compounds [1]. Knowledge of content of metals in alcoholic drinks such as wine is also very important. In many countries, alcoholic beverages, including wine, constitute more than 12 percent of the consumption of beverages in general [2]. Therefore, they could be an important source of several metal ions. Monitoring of certain elements in wines gain special attention due to their toxic effect on the human body in case of excessive intake. In fact, determination of selected metals in wine is routinely carried out in most oenological laboratories due to the fact that some elements must be kept under control according to the law (for example, according to Italian legislation, the lead content cannot exceed 0.3 mg/L) [3]. Also in other countries specific rules restricting the content of metals in wines exist, and these must be fulfilled by manufacturers to gain right to export to these markets (Table 1).

In addition, it is important to monitor the metal content in wine for authenticity purposes as well as quality control [4,5]. The elements can influence the wine making process or change its taste and quality. Moreover, metals impact on the organoleptic properties of wine [6]. A typical example can be iron, which may have a strong influence on the wine evolution during fermentation process. In fact, the importance of its impact may vary due to several reasons, for example, the coloring agents presence/absence, the aeration of the ferments level, the extent to which this element interacts with the yeast, and the aliquots which will attach to the sediments at the end of the process [7]. Copper is also an essential element, but at the same time, when consumed in excess, it is potentially toxic for humans [6]. Several elements, including Al, Cu, Fe and Zn, contribute to haze formation and taste effects [8]. Monitoring and determination of other elements, for example As, Cd and Pb, is also of high importance due to their potential toxic effects on the human body [9,10]. Besides, some metals’ content can be applied to identify its origin (vineyard and regional levels) due to the direct relationship with soil composition [11]. And although many studies were performed in this area, there are still many unanswered questions, such as:

- To what extent do processing methods affect the trace-element composition of wine?
- What happens to trace elements as wines age?
- How much inter-year variability is there in wines from one vineyard or region?

It is well known that these aspects may confound the practical issues of legal fingerprinting of wines, however, the most important monitor on composition appears to be the geographic origin of the grapes [11,12]. Three other points have occurred:

(i) multi-element dataset is needed for fingerprinting;
(ii) multivariate statistical techniques are required for data analysis; and
(iii) Inductively Coupled Plasma—Mass Spectrometry (ICP-MS) is one of the most promising analytical methods for trace and ultra-trace element fingerprinting of wines, which is characterized as fast, routine and accurate.

The present study is focused on the determination of selected metals in wines originating from different Polish vineyards and two analytical techniques were applied to gain this aim: ICP-MS and ICP-OES. In addition, chemometric analysis (Cluster analysis—CA, hierarchical and non-hierarchical with K-means algorithm, and principal components analysis—PCA) was performed to present specific correlations between the chemical variables that characterized the wine samples. Due to the fact that the metals content in wine samples impacts on the organoleptic parameters of the drink but also on human heath, information on their existence, distribution, concentration, and knowledge of existing relationships between metals and other parameters is crucial and may be useful for the food industry, health professionals, and consumers. In addition, the wine industry does not require control of the metal content in wine, thus, the knowledge of their content in this alcoholic beverage is important.

## 2. Results and Discussion

This work was intended to characterize the wine samples, origininating from different regions of Poland and made from different types of grapes, in terms of metals content. In addition, the assessment of the possible correlation between the content of metals and specific type of wines was carried out by application of chemometric tools. All parameters taken into consideration during this study are presented in Table S1.

### 2.1. Occurrence of Metals in Wine and Its Level

Thirty elements were determined by application of two techniques, ICP-OES and ICP-MS. The first technique was applied to determine Ca, K and Mg as these elements were present at high concentration levels. Information about the metal content (µg/L) in wine samples calculated as a mean (*n* = 3) is given in Table S1 (the RSD for each measurement are presented in Supplementary Materials). Almost all selected analytes were determined in wine samples, excluding Ag (not determined in 4 samples), Co (not determined in 1 sample), Cu (not determined in 7 samples), Sn (not determined in 18 samples) and V (not determined in 5 samples). The rest of the analytes were determined in all analyzed samples. However, the type of element as well as the quantity were dependent on the sample analyzed. The highest concentrations were noted for elements as follows: K (97 to 3250 mg/L), Mg (42.7 to 161 mg/L), Ca (32 to 137 mg/L), B (0.333 to 12.1 mg/L) and Mn (0.329 to 9.219 mg/L). Such elements as: Fe (0.1558 to 2.775 mg/L), Na (5.33 µg/L to 3.823 mg/L), Al (64.12 µg/L to 2.729 mg/L) and Zn (74.71 µg/L to 2.339 mg/L) occurred also at high concentration levels. The lowest concentration was noted for such metals as: Hg (0.31 to 0.51 µg/L), Ag (<0.001 to 4.92 µg/L), Co (<0.002 to 6.98 µg/L), Cd (0.023 to 2.54 µg/L), and Ti (0.54 to 2.37 µg/L). It needs to be noted that all metals content in the wine samples was much smaller than the maximum concentrations permitted according to the OIV (Table 1) or some importers of wine. A comparison of the metals in Polish wines and published data on wines of different origins is given in Table 2. However, there was no uniform tendency of metal content in given groups of wine. Therefore, it was difficult to compare those results.

Analyzing concentration levels of the metals determined, it can be concluded that depending on the color of the wine, the content of the individual element is different, for example, for silver, higher concentration levels were recorded for white wine samples than for red ones (Table 3). The same dependence can be observed for the following elements: Al, As, Bi, Cu, Sb, Se, Sn, Zr and Zn. A reverse dependence can be observed for metals such as B, Ba, Fe, K and Mn, in which higher concentration levels were found in samples of red wines. Other correlations were not visible at the first look, therefore, chemometric analysis was performed.

### 2.2. Chemometric Assessment of Multiparametric Data Set Obtained during Analysis of Wine Samples

The input dataset consisted of 44 wine (red and white) samples (objects of the study) from different regions of Poland (West Pomerania, Kyuavian Pomerania, Subcarpathia, Lesser Poland, and Masovia) and 30 metal concentrations (variables) or matrix with dimensions (44 × 30). The major goal of the chemometric assessment was to reveal specific relationships between the wine samples or between the chemical variables in order to classify the wines according to their metal content, showing in such a way which were the discriminating chemical indicators for each group of clustered wine types.

The chemometric methods used were cluster analysis (hierarchical and non-hierarchical with K-means algorithm) and principal components analysis (PCA). All three approaches are well-known and documented [14] and do not need detailed description.

**Figure 1:**
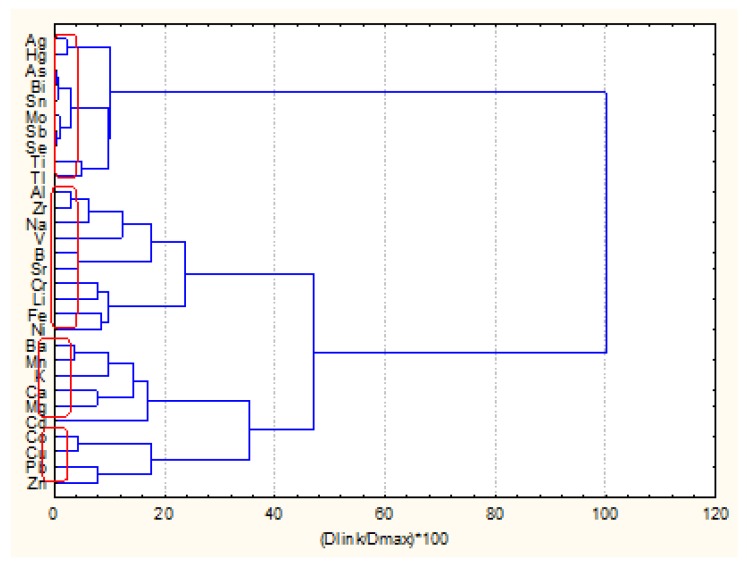
Hierarchical dendrogram for 30 chemical variables.

In Figure 1 a hierarchical dendrogram for 30 chemical variables is presented.

The HCA was performed after z-transform of the input raw data, squared Euclidean distances as similarity measures, Ward’s method of linkage and Sneath’s index for cluster significance (1/3D_max_ or 2/3 D_max_).

The unsupervised clustering led to the formation of 4 significant clusters:

K1 (Zn, Pb, Cu, Co)
K2 (Cd, Mg, Ca, K, Mn, Ba)
K3 (Ni, Fe, Li, Cr, Sr, B, V, Na, Zr, Al)
K4 (Tl, Ti, Se, Sb, Mo, Sn, Bi, As, Hg, Ag)

The next step was to perform PCA. Information on factor loadings is presented in Table 4.

Eight principal components (latent factors) explained over 80% of the total variance of the system. Pricipal component (PC)1 explained almost 27% of the total variance and showed high correlation between Ag, As, Bi, Hg, Mo, Sb, Se, Sn, Tl and Ti which corresponded entirely to K4 from HCA (conditional name “soil toxic impact”). PC2 indicated high factor loadings for *Al, V, and Zr* (subcluster of K3) and explained over 10% of the total variance (conditional name “soil specificity impact”). Nearly 10% of the total variance was explained by PC3 (Factor 5 in the Table 4; the change of order was due to the application of Varimax rotation mode) and included Ba, K, Mg, Ti (subcluster of K2) with significant factor loadings (conditional name “soil specificity impact”). PC4 (Factor 7 in the Table 4) revealed to the relationship between B, Na and Sr (another subcluster of K3) (conditional name “soil specificity impact”). Next latent factor PC5 (Factor 3 on the Table 4) explained another 9% of the total variance indicating correlation between Ca, Co, Cu and Zn resembling almost K1 (conditional name “soil specificity impact”). PC6 (Factor 4) added over 7 % to the explanation of the total variance by the high loadings of Cr, Fe, Li, Ni (another subcluster of K3) (conditional name “soil specificity impact”). The last two PCs (7 and 8 represented by Factors 6 and 8, respectively) explained each over 5% of the total variance and indicated single impacts of Cd and Pb variables (conditional name “soil toxic impact”).

PCA gave a more detailed outlook on the relationship between the chemical variables but, in general, similar relationships as in HCA were demonstrated. In general, two substantial impacts on the wine quality by the metal concentrations could be summarized: “soil toxic impact” and “soil specificity impact”.

**Figure 2:**
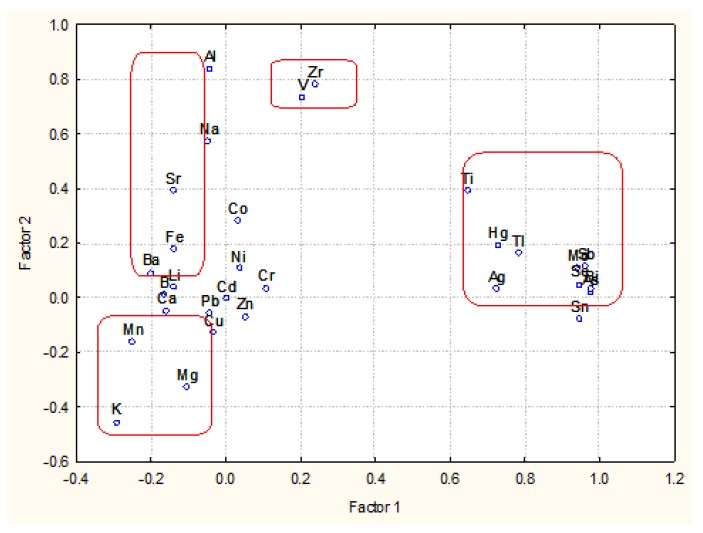
Biplot principal component (PC)1 vs. PC2.

An illustration of the relationships shows the biplot (Figure 2) where some of the links described are readily seen: Hg, Tl, Ti, Ag or K, Mg, Mn. It should be kept in mind that this blot illustrate only 37% of the total variance and one cannot expect to see all relationships valid for 8 PCs.

**Figure 3:**
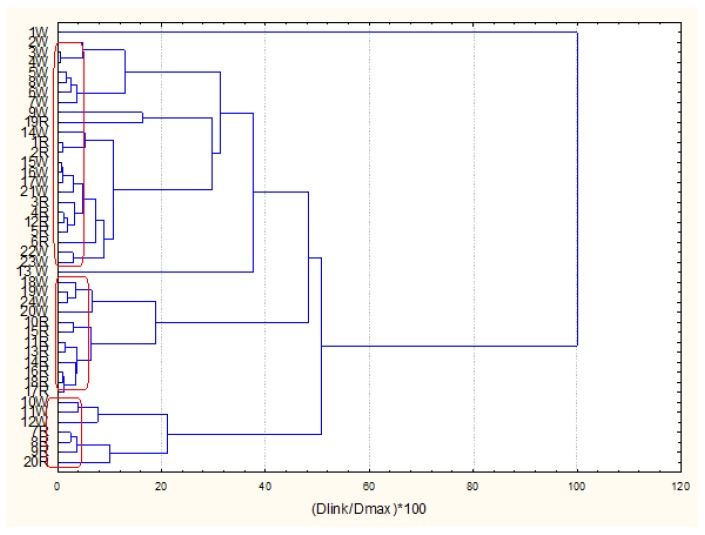
Hierarchical dendrogram for 44 wine samples.

Since the more significant task was to detect groups of similarity between the wine samples, the next figure shows the hierarchical dendrogram for all 44 samples. In Figure 3 three significant clusters were formed as follows:

K1 (**10w, 11w, 12w**, 7r, 8r, 9r, 20r)
K2 (**18w, 19w, 20w, 24w**, 10r, 11r, 13r, 14r, 15r, 16r, 17r, 18r)
K3 (2w, 3w, 4w, 5w, 6w, 7w, 8w, 9w, 13w, 14w, 15w, 16w, 17w, 21w, 22w, 23w, 1r, 2r, 3r, 4r, 5r, 6r, 12r, 19r)

***1w*** was an outlier

It could be concluded that K1 was a smaller cluster consisting of an almost equal number of white and red wines; K2 was dominated by red wines in a ratio of 2:1 to the white wines and K3 was dominated by white wines in a ratio of 2:1 to the red wines. This separation was with respect to the region of origin of the wines. Thus, K1 included the Subcarpathia wines (red and white), K2 was the cluster of mostly wines from Masovia and K3 involved wines from West Pomerania, Pomerania and Kyuavian Pomerania.

**Figure 4:**
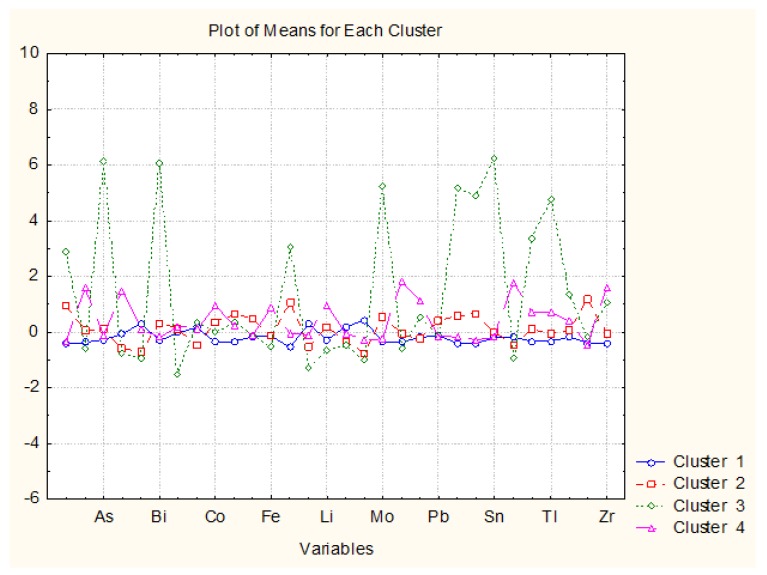
Plot of average values of the chemical concentrations of metals in each of the identified clusters.

The next important issue was to determine the discriminant chemical variables (indicated in this case by the metal concentrations) for each one of the identified clusters. A very useful approach is the supervised classification by the use of K-means non-hierarchical cluster method, where the number of clusters could be predetermined. A plot of average values of the chemical concentrations of metals in each of the identified clusters is presented in Figure 4.

The clustering of the objects by K-means in 4 predetermined clusters confirmed entirely the results of the hierarchical clustering of the wine samples. There were obtained 1 outlier and three clusters having the same content of objects (wine samples) as indicated above.

Wine sample ***1w*** (Cluster 3, different from all others, outlier) was characterized by the highest levels of Ag, As, Bi, Hg, Mo, Sb, Se, Sn, Ti, Tl (the group of metals having high factor scores in PC1 which could be conditionally named “toxicated” by soil conditions, probably resulting from a different local pollution impact for this specific location as compared to all other Pomeranian locations) and lowest levels of Al, V, Zr (elements from PC2), B, Ba, K, Mg, Mn (PC3), that is, earth constituents or “specific” soil conditions). No other West Pomeranian wine showed similar properties.

Cluster 1 (the same as K3 in HCA) consisted of all different Pomeranian wines; it was characterized by “background” levels of all metals and did not have specific discriminating species. It might mean that the soil conditions were very appropriate for the grapes ensuring high wine quality.

Cluster 2 (the same as K2 in HCA) consisted of Masovian wines and obviously both white and red products resembled the quality of the Pomeranian wines—good soil conditions (no “toxic” or “specific” soil conditions impacting the wine quality).

Cluster 4 (the same as K1in HCA) consisted of Subcarpathian wines which were obviously subject to soil specificity as they resembled more the outlier wine type rather than the other two types (Masovian and Pomeranian). These samples were characterized by enhanced levels of Al, B, Co, Fe, Li, Na, Ni, Sr, Zr, thus becoming subject to soil specificity (of natural, nor anthropogenic origin) in the region.

## 3. Materials and Methods 

### 3.1. Reagents and Standards

For calibration of ICP-MS the ICP IV multi element standard was used (Merck, New York, NY, USA) and single standards: As, Sb, Se, Mo and V (Sigma-Aldrich, New York, NY, USA), Hg (Merck, USA). Moreover, as an internal standards Sc, Rh, Tb and Ge in supra pure 1% HNO_3_ (Merck, New York, NY, USA) and deionized water obtained from the Milli-Q Direct 8 Water Purification System (Merck Millipore, New York, NY, USA) were used for sample (pre)treatment and sample dilution. For calibration of ICP-OES the Sigma Aldrich (USA) stock solution containing 1000 mg L^−1^ of Ca, Mg, and K were used to prepare calibration standards. The accuracy of the analyses was verified by means of certified reference materials: trace metals ICP—sample 1 and trace metals ICP—sample 2. The retrieval of the selected elements in both CRMs ranged from 97% to 105%. At the same time, water and matrix calibration standards were compared (in 1.2% C_2_H_5_OH) and no significant differences were found in the obtained values of the analytical signals from the both standards type.

### 3.2. Samples

Fourty-four bottles of wine, made from different kinds of grapes, were obtained from 9 vineyards located in different parts of Poland: Zodiak Vineyard (West Pomerania), PrzyTalerzyku Vineyard and Kozielec Vineyard (Kuyavian-Pomerania), Pod OrzechemVienyard (Pomerania), Stok Vineyard (Masovia), Spotkaniówka Vineyard and Dwór Kombornia Vineyard (Subcarpathia), Chodorowa Vineyard and UJ Vineyard (Lesser Poland). These vineyards differ in terms of the type of soil and height above the sea, which results in a different climate. The emphasis was placed on collecting wines made from different variety of grapes from a large number of geographically distinct vineyards representing the six main regions noted above. Altogether there were 24 white wines and 20 white wines in this study. Most wines were made from a single grape variety with >13 varieties represented; some of samples were cuvee. Some of the wines are available on an industrial scale, while the next group of samples are regional wines which are not under any control.

Samples of wines were diluted 10 times with deionized water, without any prior preparation.

### 3.3. Equipment Used

Concentration of Ca, K, Mg was determined by the ICP-OES (Shimadzu ICPE-9820, Japan) analytical technique with application of a published previously procedure [15]. The samples were introduced into the plasma, using a high-accuracy nebulizer by free aspiration and a gravity drain. Emission lines were selected based on their sensitivity. The ionic lines of Ca II (393.366 nm), Mg II (279.553 nm) and K I (766.490 nm) were measured. Information on the operating conditions for the multielemental analysis of wines on an ICP-OES spectrometer are presented in Table 5.

ICP-MS analytical technique (ICP-MS 2030 Shimadzu, Japan) was applied to determine and quantify most of the metals in wine samples. Table 5 represents information on the operating conditions for the wine analysis on an ICP-MS spectrometer.

The limit of detection (LOD) values were calculated as the concentrations corresponding to signals equaled to three times the standard deviation of blank solution signal, both for 10 and 7.0 L min−1 plasma flow rate [15].

Table S2 (See Supplementary Materials) presents calibration curve values as well as LOD and LOQ values. Moreover Table S3 contain validation report of the ICP-MS analytical technique is presented.

### 3.4. Chemometric Analysis

One of the most used chemometric methods for multivariate data interpretation is cluster analysis (CA, hierarchical and non-hierarchical clustering) [14] and this tool was applied in this study. Cluster analysis is a well-known multivariate statistical method which makes it possible to detect spontaneous similarity patterns (clusters) within a large data set or to prove a priori a given hypothesis of n-number of existing patterns in the set. The first approach required hierarchical clustering and the second non-hierarchical clustering. Both modes are well documented and do not need detailed explanation [14]. In general, data standardization, similarity determination and linkage procedures are applied to reach a solution.

In the case of missing data (under limit of detection), the value of LOD/2 was introduced. The software package applied was STATISTICA 8.0 (New York, NY, USA).

## 4. Conclusions

This study presents the results of the determination of 30 metals in Polish wines samples originating from several vineyards located in different regions of Poland. Almost all analytes selected were determined in wine samples, excluding Ag, Co, Cu, Sn, and V. However, the type of element, as well as the quantity, depended on the sample analyzed. Data obtained showed that none of the wine samples surpassed the toxic levels of metals reported in the literature. Thus, these wines appeared to be safe as regards the risk associated with the potentially toxic metals intake. However, analyzing concentration levels of metals, it can be concluded that depending on the color of the wine, the content of the individual element was different, for example, for silver, higher concentration levels were recorded for white wine samples than for red ones. Other correlations were not visible at the first look. Therefore the chemometric analysis was performed, which revealed that two major factors are responsible for the wine quality—soil toxic metals content and soil natural composition. The wine samples could be readily separated by geographic objectives, and for each region some discriminating metal variables could be defined. Further, the data structure should be studied to indicate possible sources of the metal content (natural and anthropogenic). In addition, the application of stable isotope analysis of hydrogen and oxygen would be effective to survey the origin of wines.

## Figures and Tables

**Table 1:** The accepted limits of metals content (mg/L) in wine in different countries and given by OIV.

Country	Concentration of Metals (mg/L)							
	Al	As	Cd	Cu	Na	Pb	Ti	Zn
Australia	-	0.10	0.05	5.00	-	0.20	-	5.00
Germany	8.00	0.10	0.01	5.00	-	0.30	1.00	5.00
Italy	-	-	-	10.00	-	0.30	-	5.00
Poland	-	0.20	0.03	-	-	0.30	-	-
OIV	-	0.20	0.01	1.00	60	0.15	-	5.00

**Table 2:** Comparison of the metals concentration of wines of different origins [13].

Metal		Concentration of Metals (mg/L) in Wine of Different Origin								
	American	Czech	Ethiopian	French	German	Greek	Hungarian	Italian	Spanish	Polish
K	462–1147	493–3056	694–767	265–426	480–1860	955–2089	489–1512	750–1500	338–2032	97–3250
Ca	17–94	40–100	28–37	65–161	58–200	14.0–47.5	51–164	30–151	12–241	32–137
Mg	100–245	7.8–138	58–79	55–96	56–105	82.5–122.5	72–174	53–115	50–236	42.7–161
Pb	--	0.010–1.253	0.14–031	0.006–0.023	--	ND–0.62	--	0.01–0.35	0.001–0.096	0.006–0.349
Zn	0.75–3.60	--	1.82–2.70	0.44–0.74	0.3–1.5	0.05–8.9	0.6–1.9	0.135–4.8	ND–4.63	0.007–2.339
Cd	--	0.000055–0.0033	<0.01	ND–0.0002	--	ND–0.03	0.00014–0.54	0.0012–0.0016	ND–0.019	0.00002–0.0025
Fe	1.2–6.6	0.9–5.2	1.42–3.16	0.81–2.51	0.4–4.2	0.7–7.3	2.03–23.7	1.35–27.8	0.4–17.4	0.1558–2.775
Na	7–106	2.0–110	24–25	7.7–14.6	6–25	5.5–150	18.6–81.1	3.4–200	3.5–300	0.005–3.823
Cu	0.05–0.58	0.012–6.827	0.5–1.5	ND–0.48	0.02–0.71	0.2–1.65	0.15–2.57	0.001–1.34	ND–3.1	<0.0002–0.796
Mn	0.81–4.08	0.28–3.26	1.04–1.88	0.63–0.96	0.5–1.3	ND–2.3	0.12–2.9	0.67–2.5	0.1–5.5	0.329–9.219
Ni	--	0.19–0.034	0.18–0.25	ND–0.052	--	ND–0.5	--	0.015–0.21	0.005–0.079	0.00002–0.245
Co	--	ND–0.018	ND–0.09	0.004-0.005	0.004-0.005	ND–0.04	0.003–0.009	0.003-0.006	ND–04	<0.00002–0.007
Cr	--	0.032–0.037	ND–0.09	0.006-0.09	0.01-0.41	ND–0.41	0.032–0.062	0.02-0.05	0.025–0.029	0.0037–0.095

**Table 3:** The average concentration of each metal content in white and red Polish wines.

Type of Wine	Concentration of Metal																													
	Ag	Al	As	Ba	Bi	Cd	Co	Cr	Cu	Fe	Hg	Li	Mo	Na	Ni	Pb	Sb	Se	Sn	Sr	Ti	Tl	V	Zr	B	Ca	K	Mg	Mn	Zn
	[µg/L]																								[mg/L]					
W	1.4	710	26	140	49	1	2	14	290	520	0.4	5,6	7.4	890	64	20	2.3	24	14.1	290	33	0.87	7.5	5.9	3730	7461	91145	9376	192	73.4
R	0.2	580	6	310	2	0,6	2	12	130	790	0.3	11.2	3.4	1050	53	28	0.4	3.9	0.12	490	30	0.81	8.2	2.5	6870	7504	182845	10600	318	34.8

**Table 4:** Information on factor loadings.

Variable	PC1	PC2	PC3	PC4	PC5	PC6	PC7	PC8
	Factor 1	Factor 2	Factor 3	Factor 4	Factor 5	Factor 6	Factor 7	Factor 8
**Ag**	0.728	0.030	−0.149	−0.007	0.324	0.048	−0.193	−0.153
**Al**	−0.041	0.835	−0.021	0.232	0.113	−0.046	0.248	0.015
**As**	0.976	0.018	0.069	−0.026	0.005	0.005	0.016	0.033
**B**	−0.162	0.007	0.097	0.217	−0.196	0.065	0.836	0.062
**Ba**	−0.199	0.089	0.147	−0.185	−0.842	0.067	0.164	−0.063
**Bi**	0.980	0.030	0.060	−0.005	0.051	−0.014	−0.061	−0.002
**Ca**	−0.158	−0.051	−0.679	0.228	−0.457	0.068	−0.105	−0.074
**Cd**	0.003	−0.008	0.033	−0.127	−0.134	−0.903	−0.045	−0.024
**Co**	0.032	0.281	−0.837	−0.080	0.197	−0.127	0.193	−0.093
**Cr**	0.111	0.033	−0.130	0.856	0.105	0.021	−0.130	0.079
**Cu**	−0.032	−0.130	−0.852	−0.183	0.107	0.108	−0.087	0.082
**Fe**	−0.136	0.176	0.076	0.646	−0.258	0.232	0.237	0.154
**Hg**	0.733	0.187	−0.319	0.040	0.386	−0.087	−0.256	−0.131
**K**	−0.290	−0.458	−0.042	0.018	−0.508	0.348	0.307	−0.040
**Li**	−0.135	0.034	0.029	0.517	0.275	0.112	0.406	0.483
**Mg**	−0.100	−0.331	−0.356	0.096	−0.531	−0.214	0.011	−0.323
**Mn**	−0.246	−0.165	0.185	−0.107	−0.775	−0.395	0.017	0.017
**Mo**	0.944	0.109	0.035	0.083	0.062	0.008	−0.165	0.009
**Na**	−0.045	0.570	−0.055	0.052	0.243	−0.083	0.627	0.014
**Ni**	0.037	0.104	0.177	0.638	0.164	−0.028	0.309	−0.043
**Pb**	−0.041	−0.062	0.006	0.088	0.007	−0.003	0.016	0.927
**Sb**	0.965	0.112	0.022	0.033	0.128	-0.008	−0.130	0.033
**Se**	0.948	0.047	0.013	0.057	0.155	0.032	−0.152	0.008
**Sn**	0.947	−0.079	0.071	−0.079	−0.051	0.064	0.060	0.051
**Sr**	−0.138	0.390	−0.014	0.099	−0.355	0.041	0.711	−0.102
**Ti**	0.648	0.388	−0.008	0.030	0.215	−0.408	0.181	−0.160
**Tl**	0.785	0.160	0.068	−0.115	0.048	-0.136	0.455	−0.060
**V**	0.206	0.729	0.122	−0.092	−0.134	0.242	−0.138	−0.029
**Zn**	0.053	−0.071	−0.587	0.191	0.228	0.053	−0.314	0.530
**Zr**	0.242	0.776	−0.123	0.163	0.097	−0.138	0.275	−0.093
**Expl.Var %**	26.9	10.2	8.9	7.4	9.9	5.1	9.3	5.5

**Table 5:** Information on inductively coupled plasma-mass spectrometry (ICP-MS) 2030 and inductively coupled plasma-optical emission spectrometry (ICP-OES) 9820 measurement conditions/parameters and limit of detection for each element.

Parameter and Accessories	ICP-MS	ICP-OES
Radio frequency power generator [kW]	1.2	1.2
Gas type	Argon	Argon
Plasma gas flow rate [L min^−1^]	8.0	7.0
Auxiliary gas flow rate [L min^−1^]	1.1	0.6
Nebulization gas flow rate [L min^−1^]	0.7	0.7
Torch	Mini-torch (quartz)	Mini-torch (quartz)
Nebulizer	Coaxial	Burgener NX-175
Spray chamber temperature	3 °C	Room temperature
Drain	Gravity fed	Gravity fed
Internal Standard	Automatic addition	-
Sampling depth	5 mm	-
Collision Cell Gas flow (He)	6 mL min^−1^	-
Cell Voltage	−21 V	-
Enersgy Filter	7.0 V	-
Number of replicates	3	3
Integration conditions/number of scans	10	-

## Supplementary Materials

The following are available online.
