# Supplementary material for: Determination of Metals Content in Wine Samples by Inductively Coupled Plasma-Mass Spectrometry
Source: Molecules. 2018 Nov 5;23(11):2886. doi: 10.3390/molecules23112886 (PMC6278765; doi:10.3390/molecules23112886)
Supplement: Supplementary file 1 [file molecules-23-02886-s001.pdf]

molecules

MDPI

Table S1. Results of the metals content in wine samples ($n = 3$; $k = 2$, $\alpha = 0.05$).

No_{sample}	Grape Type	Region of Poland	Location [Masl]	Color	Metal Content (µg/L) or (mg/L) /Isotope Mass (amu)																													
					[µg/L]																								[mg/L]					
					Ag 107	Al 27	As 75	Zr 90	Ba 138	Bi 209	Cd 111	Co 59	Cr 52	Cu 63	Fe 56	Hg 201	V 51	Li 7	Mo 98	Na 23	Ni 60	Pb 206	Sb 121	Se 78	Sn 115	Sr 88	Ti 47	Tl 205	Mg 25	K 41	Mn 55	B 10	Zn 64	Ca 40
1W	Solaris	W P	125	w	4.92 ± 0.15	206.1 ± 7.8	290.5 ± 28	10.91 ± 0.40	65.62 ± 1.12	456.1 ± 12.3	1.05 ± 0.16	2.05 ± 0.30	16.32 ± 0.75	396.2 ± 11.8	424.1 ± 12.0	0.51 ± 0.091	15.31 ± 0.45	1.68 ± 0.20	39.11 ± 1.13	170.3 ± 7.8	87.21 ± 1.47	23.51 ± 0.43	13.21 ± 0.34	139.1 ± 2.4	200.2 ± 7.9	126.1 ± 2.4	46.91 ± 1.1	2.37 ± 0.31	84.8 ± 1.5	422 ± 12	0.506 ± 0.091	2.94 ± 0.17	4.95 ± 0.30	43.4 ± 1.1
2 W	Solaris	W P	125	w	4.41 ± 0.16	64.12 ± 1.12	92.71 ± 2.9	5.81 ± 0.30	118.1 ± 2.3	118.7 ± 2.0	0.56 ± 0.091	1.61 ± 0.26	10.31 ± 0.43	394.3 ± 11.5	390.6 ± 11.8	0.47 ± 0.090	6.18 ± 0.37	1.41 ± 0.18	18.61 ± 0.50	333.3 ± 10.2	80.72 ± 1.51	22.22 ± 0.45	6.89 ± 0.22	88.03 ± 1.51	43.7 ± 1.1	142.2 ± 2.5	36.72 ± 1.12	1.08 ± 0.13	64.1 ± 1.3	591±14	0.484±0.089	2.73±0.19	5.77±0.31	79.6±1.8
3 W	Seyval Blanc	W P	125	w	4.21 ± 0.12	1511 ± 12	38.42 ± 0.54	8.33 ± 0.39	136.5 ± 2.5	85.77 ± 1.71	0.66 ± 0.096	1.15 ± 0.20	4.94 ± 0.22	53.95 ± 0.54	357.2 ± 11.6	0.45 ± 0.087	8.26 ± 0.42	2.91 ± 0.27	12.51 ± 0.43	1632 ± 25	16.51 ± 1.12	16.22 ± 0.37	4.84 ± 0.21	49.31 ± 1.32	29.9 ± 0.56	355.1 ± 6.4	34.63 ± 1.15	1.11 ± 0.10	65 ± 1.3	600 ± 15	1.26 ± 0.20	2.34 ± 0.18	2.02 ± 0.23	80.2 ± 1.9
4 W	Seyval Blanc	W P	125	w	4.29 ± 0.17	1608 ± 13	24.24 ± 0.43	7.49 ± 0.36	137.5 ± 2.4	70.98 ± 1.53	0.65 ± 0.094	1.12 ± 0.21	4.87 ± 0.23	54.41 ± 0.54	351.3 ± 11.4	0.43 ± 0.086	7.03 ± 0.40	2.83 ± 0.29	9.87 ± 0.30	1633 ± 27	18.22 ± 1.19	15.77 ± 0.34	3.85 ± 0.20	38.91 ± 1.01	18.2 ± 0.34	354.5 ± 6.2	34.71 ± 1.14	0.98 ± 0.023	84 ± 1.5	757 ± 17	1.28 ± 0.21	2.45 ± 0.16	2.18 ± 0.21	59.7 ± 1.6
5 W	Bianca	KP	74	w	2.35± 0.10	145.2 ± 5.1	14.31 ± 0.21	2.14 ± 0.23	75.64 ± 1.31	51.91 ± 1.21	0.51 ± 0.091	1.67± 0.26	12.11 ± 0.56	19.11 ± 0.34	613.2 ± 15.8	0.43 ± 0.082	2.77 ± 0.23	4.73 ± 0.31	7.41 ± 0.32	305.5 ± 10.5	29.61 ± 1.25	18.11 ± 0.38	2.61 ± 0.19	25.71 ± 1.02	7.54 ± 0.45	239.6 ± 7.9	28.61 ± 1.11	0.81 ± 0.023	135 ± 1.8	733 ± 18	0.940 ± 0.18	3.05 ± 0.21	8.73 ± 0.37	44.2 ± 1.2

**No$_{sample}$**	**Grape Type**	**Region of Poland**	**Location [Masl]**	**Color**	**Metal Content (µg/L) or (mg/L) /Isotope Mass (amu)**																													
					**[µg/L]**																								**[mg/L]**					
					**Ag 107**	**Al 27**	**As 75**	**Zr 90**	**Ba 138**	**Bi 209**	**Cd 111**	**Co 59**	**Cr 52**	**Cu 63**	**Fe 56**	**Hg 201**	**V 51**	**Li 7**	**Mo 98**	**Na 23**	**Ni 60**	**Pb 206**	**Sb 121**	**Se 78**	**Sn 115**	**Sr 88**	**Ti 47**	**Tl 205**	**Mg 25**	**K 41**	**Mn 55**	**B 10**	**Zn 64**	**Ca 40**
6 W	Solaris	KP	74	w	1.840 ± 0.091	170.5 ± 5.8	19.83 ± 0.27	1.73 ± 0.19	65.51 ± 1.12	40.71 ± 1.12	0.66 ± 0.097	3.76 ± 0.34	16.11 ± 0.75	61.71 ± 0.59	855.1 ± 23.8	0.42 ± 0.085	2.14 ± 0.21	4.44 ± 0.33	5.58 ± 0.31	338.6 ± 10.2	61.72 ± 1.23	10.61 ± 0.32	2.38 ± 0.19	47.12 ± 1.51	6.69 ± 0.43	172.7±7.5	31.82 ± 1.10	0.86 ± 0.024	69.5 ± 1.4	828 ± 18	1.14 ± 0.20	4.42 ± 0.24	1.29 ± 0.19	88.3 ± 2.1
7 W	Aurora. Bianca Hibernal. Muscat	P	120	w	1.520 ± 0.089	906.1 ± 11.1	15.87 ± 0.25	5.51 ± 0.30	92.11 ± 1.71	48.62 ± 1.14	0.41 ± 0.081	3.79 ± 0.35	24.81 ± 0.87	20.62 ± 0.33	637.2 ± 20.8	0.42 ± 0.083	17.91 ± 0.45	6.09 ± 0.45	15.31 ± 0.43	791.7 ± 20.1	39.91 ± 1.34	13.5 ± 0.35	2.56 ± 0.20	26.55 ± 0.87	6.97 ± 0.43	321.1 ± 8.2	33.11 ± 1.15	0.65 ± 0.021	93.1 ± 1.8	890 ± 19	3.48 ± 0.34	3.32 ± 0.18	2.21 ± 0.23	85.5 ± 1.9
8 W	Aurora. Bianca	P	120	w	1.390 ± 0.072	327.1 ± 8.8	13.02 ± 0.19	3.97 ± 0.26	64.83 ± 1.15	48.33 ± 1.19	0.27 ± 0.034	1.21 ± 0.19	22.84 ± 0.89	15.93 ± 0.31	482.4 ± 12.1	0.41 ± 0.084	8.52 ± 0.36	3.01 ± 0.32	8.47 ± 0.32	493.1 ± 13.1	43.25 ± 1.33	12.29 ± 0.27	2.15 ± 0.19	24.66 ± 0.89	4.17 ± 0.45	165.1 ± 7.1	31.84 ± 1.12	0.58 ± 0.018	100 ± 1.9	965 ± 20	0.329 ± 0.078	2.96 ± 0.16	1.55 ± 0.20	72.5 ± 1.8
9 W	Hibernal. Bianca. Muller Turgau	KP	92	w	1.660 ± 0.088	577.1 ± 14.2	8.83 ± 0.17	1.96 ± 0.17	63.02 ± 1.17	34.92 ± 1.10	0.36 ± 0.039	1.08 ± 0.19	95.17 ± 1.23	769.1 ± 11.9	1324 ± 28	0.39 ± 0.079	2.77 ± 0.21	27.21 ± 1.0	7.38 ± 0.29	345.2 ± 10.4	174.7 ± 2.5	48.69 ± 1.12	1.86 ± 0.16	25.11 ± 0.83	2.33 ± 0.23	222.1 ± 7.9	30.18 ± 1.11	0.58 ± 0.018	115 ± 2.0	1410 ± 23	1.40 ± 0.22	4.65 ± 0.23	1.42 ± 0.17	95.2 ± 2.2
10 W	Bianca	SC	320	w	1.170 ± 0.064	2059 ± 32	11.71 ± 0.19	25.42 ± 0.89	261.2 ± 4.9	33.85 ± 1.09	0.94 ± 0.19	5.47 ± 0.40	11.35 ± 0.50	63.44 ± 0.58	475.5 ± 11.9	0.38 ± 0.077	4.44 ± 0.24	9.57 ± 0.89	4.41 ± 0.26	3732 ± 39	78.27 ± 1.51	21.71 ± 0.54	1.73 ± 0.15	15.62 ± 0.65	3.11 ± 0.25	951.1 ± 11.2	38.99 ± 1.16	1.26 ± 0.23	104 ± 1.9	880 ± 20	1.42 ± 0.22	5.52 ± 0.27	3.50 ± 0.25	76.7 ± 1.9

No_{sample}	Grape Type	Region of Poland	Location [Masl]	Color	Metal Content (µg/L) or (mg/L) /Isotope Mass (amu)																													
					[µg/L]																								[mg/L]					
					**Ag 107**	**Al 27**	**As 75**	**Zr 90**	**Ba 138**	**Bi 209**	**Cd 111**	**Co 59**	**Cr 52**	**Cu 63**	**Fe 56**	**Hg 201**	**V 51**	**Li 7**	**Mo 98**	**Na 23**	**Ni 60**	**Pb 206**	**Sb 121**	**Se 78**	**Sn 115**	**Sr 88**	**Ti 47**	**Tl 205**	**Mg 25**	**K 41**	**Mn 55**	**B 10**	**Zn 64**	**Ca 40**
11 W	Hibernal	SC	320	w	0.73 ± 0.056	1174 ± 20	12.97 ± 0.23	7.88 ± 0.39	190.4 ± 4.2	24.25 ± 1.08	1.56 ± 0.23	3.64 ± 0.34	13.41 ± 0.59	155.5 ± 2.3	456.2 ± 11.7	0.41 ± 0.089	4.75 ± 0.26	16.64 ± 1.20	5.35 ± 0.31	3823 ± 41	69.56 ± 1.43	27.85 ± 0.32	1.84 ± 0.17	12.82 ± 0.43	3.03 ± 0.25	401.4 ± 8.2	38.67 ± 1.15	0.97 ± 0.021	94.1 ± 1.7	1160 ± 21	1.49 ± 0.24	7.67 ± 0.32	3.89 ± 0.25	90.8 ± 2.1
12 W	Hibernal	SC	320	w	0.48 ± 0.055	2729 ± 22	22.75 ± 0.22	33.21 ± 0.78	182.1 ± 4.0	20.36 ± 1.11	0.89 ± 0.16	4.97 ± 0.39	24.83	126.1 ± 1.8	816.9 ± 25.8	0.39 ± 0.077	25.89 ± 0.49	13.02 ± 0.78	4.87 ± 0.30	3421 ± 42	142.9 ± 2.4	26.47 ± 0.24	1.57 ± 0.14	11.47 ± 0.39	1.56 ± 0.13	867.7 ± 12.2	43.41 ± 1.19	1.03 ± 0.029	92.1 ± 1.8	188 ± 2.3	1.41 ± 0.21	9.93 ± 0.36	5.71 ± 0.30	75.5 ± 1.8
13 W	Hibernal	SC	296	w	1.840 ± 0.091	56.66 ± 1.91	3.11 ± 0.10	1.21 ± 0.12	43.31 ± 1.09	1.71 ± 0.091	0.51 ± 0.091	<0.002	7.12 ± 0.27	25000 ± 29	155.8 ± 2.8	0.43 ± 0.091	0.37 ± 0.14	1.13 ± 0.20	1.01 ± 0.19	99.94 ± 1.81	0.21 ± 0.012	23.24 ± 0.30	0.56 ± 0.012	6.79 ± 0.37	<0.021	49.7 ± 1.2	24.61 ± 1.10	0.54 ± 0.017	123 ± 2.2	1540 ± 23	0.018 ± 0.007	0.333 ± 0.019	2.34 ± 0.21	137
14 W	Hibernal	LP	335	w	0.590 ± 0.046	1127 ± 19	9.41 ± 0.24	2.22 ± 0.19	187.1 ± 4.3	24.51 ± 1.10	0.44 ± 0.078	2.86 ± 0.32	13.61 ± 0.56	98.21 ± 0.98	331.8 ± 4.5	0.37 ± 0.081	20.19 ± 0.47	8.69 ± 0.67	5.99 ± 0.27	2491 ± 29	23.11 ± 1.12	18.16 ± 0.21	1.06 ± 0.021	6.93 ± 0.40	2.22 ± 0.22	503.4 ± 4.2	31.02 ± 1.14	1.15 ± 0.23	42.7 ± 1.3	1130 ± 21	0.99 ± 0.17	5.89 ± 0.34	9.89 ± 0.67	42.9 ± 1.3
15 W	Jutrzenka	M	151	w	0.610 ± 0.041	387.1 ± 9.2	5.75 ± 0.28	3.81 ± 0.28	202.4 ± 4.2	25.31 ± 1.09	0.65 ± 0.095	1.26 ± 0.26	6.11 ± 0.22	436.1 ± 11.5	407.1 ± 11.8	0.38 ± 0.080	7.5 ± 0.34	2.47 ± 0.28	2.73 ± 0.23	169.3 ± 7.8	50.91 ± 1.32	43.85 ± 0.43	1.79 ± 0.11	10.81 ± 0.32	2.71 ± 0.23	150.6 ± 5.1	29.12 ± 1.11	0.67 ± 0.020	84.2 ± 1.5	1090 ± 20	1.57 ± 0.25	1.44 ± 0.17	5.66 ± 0.30	72.4 ± 1.8
16 W	Jutrzenka	M	151	w	0.390 ± 0.039	427.1 ± 9.6	3.47 ± 0.19	1.55 ± 0.18	174.4 ± 3.7	20.92 ± 1.11	0.51 ± 0.091	1.36 ± 0.27	4.86 ± 0.22	29.47 ± 0.34	680.3 ± 23.8	0.35 ± 0.067	1.67 ± 0.11	3.65 ± 0.32	4.57 ± 0.32	110.4 ± 3.2	10.19 ± 1.01	6.14 ± 0.29	0.87 ± 0.021	5.56 ± 0.25	1.58 ± 0.18	143.1 ± 2.4	26.82 ± 1.10	0.65 ± 0.021	88.9 ± 1.6	817 ± 19	2.26 ± 0.27	2.57 ± 0.19	3.05 ± 0.25	78.5 ± 1.6

No$_{sample}$	Grape Type	Region of Poland	Location [Masl]	Color	Metal Content (µg/L) or (mg/L) /Isotope Mass (amu)																											
					[µg/L]																						[mg/L]					
					Ag 107	Al 27	As 75	Zr 90	Ba 138	Bi 209	Cd 111	Co 59	Cr 52	Cu 63	Fe 56	Hg 201	V 51	Li 7	Mo 98	Na 23	Ni 60	Pb 206	Sb 121	Se 78	Sn 115	Sr 88	Ti 47	Tl 205	Mg 25	K 41	Mn 55	B 10	Zn 64	Ca 40
17 W	Aurora. Bianca	M	151	w	0.39 ± 0.038	342.1 ± 9.1	5.84 ± 0.33	3.11 ± 0.23	181.4 ± 4.1	17.11 ± 1.08	1.21 ± 0.21 ± 0.034	2.12 ± 0.31	11.77 ± 0.50	127.5 ± 1.7	761.2 ± 23.9	0.36 ± 0.078	8.76 ± 0.31	4.16 ± 0.34	2.27 ± 0.23	480.1 ± 10.1	62.38 ± 1.51	19.61 ± 0.33	0.98 ± 0.019	11.72 ± 0.43	1.37 ± 0.12	282.1 ± 7.9	27.42 ± 1.00	0.55 ± 0.018	106 ± 1.9	862 ± 14	2.07 ± 0.24	1.88 ± 0.16	3.28 ± 0.21	87.2 ± 1.9
18 W	Aurora. Bianca	M	151	w	0.31 ± 0.037	232.1 ± 7.8	4.11 ± 0.22	1.48 ± 0.11	117.3 ± 2.3	14.41 ± 1.07	1.09 ± 0.16	1.46 ± 0.27	8.63 ± 0.26	<0.268	290.2 ± 3.2	0.36 ± 0.075	7.46 ± 0.27	2.74 ± 0.29	2.04 ± 0.22	33.63 ± 0.69	35.71 ± 1.41	13.91 ± 0.22	0.78 ± 0.016	5.78 ± 0.21	0.84 ± 0.023	279.2 ± 7.7	30.12 ± 1.13	0.6 ± 0.019	161 ± 2.5	1390 ± 24	2.45 ± 0.27	3.24 ± 0.22	3.81 ± 0.26	122
19 W	La Crescent	M	151	w	0.56 ± 0.041	402.2 ± 9.8	3.91 ± 0.19	1.41 ± 0.10	230.7 ± 4.7	14.27 ± 1.01	2.11 ± 0.30	2.82 ± 0.35	6.77 ± 0.31	226.1 ± 3.2	476.1 ± 11.5	0.36 ± 0.075	1.92 ± 0.11	1.03 ± 0.20	5.03 ± 0.29	77.74 ± 1.13	21.41 ± 1.14	6.21 ± 0.23	0.61 ± 0.014	3.94 ± 0.19	0.87 ± 0.045	280.2 ± 7.4	33.71 ± 1.15	1.06 ± 0.13	156 ± 2.5	985 ± 19	7.70 ± 0.48	4.00 ± 0.20	4.77 ± 0.29	95.4 ± 2.2
20 W	La Crescent. St. Pepin	M	151	w	0.29 ± 0.028	226.2 ± 7.6	4.04 ± 0.20	1.18 ± 0.10	186.4 ± 4.2	10.68 ± 1.02	3.73 ± 0.36	2.12 ± 0.32	5.46 ± 0.27	164.4 ± 2.8	361.2 ± 2.9	0.35 ± 0.072	0.88 ± 0.023	0.58 ± 0.14	2.99 ± 0.24	39.29 ± 0.39	32.61 ± 1.31	6.87 ± 0.19	0.51 ± 0.013	4.99 ± 0.32	0.72 ± 0.046	289.1 ± 7.4	31.51 ± 1.12	0.87 ± 0.016	69.3 ± 1.5	901 ± 17	6.84 ± 0.46	3.97 ± 0.21	6.65 ± 0.33	55 ± 1.5
21 W	Andalamina. Kristally. Prarie Star	M	151	w	0.13 ± 0.006	559.9 ± 11.8	6.12 ± 0.26	3.97 ± 0.27	156.1 ± 3.2	7.19 ± 0.67	0.91 ± 0.15	1.04 ± 0.15	4.75 ± 0.22	47.31 ± 0.50	469.3 ± 11.9	0.35 ± 0.073	19.77 ± 0.46	3.07 ± 0.29	4.61 ± 0.36	188.9 ± 7.8	33.84 ± 1.36	13.81 ± 0.34	0.62 ± 0.016	10.11 ± 0.87	0.57 ± 0.032	170.5 ± 6.2	27.95 ± 1.11	0.61 ± 0.014	62.5±1.4	166 ± 2	2.46 ± 0.29	2.30 ± 0.17	2.54 ± 0.21	43.9 ± 1.3
22 W	Andalamina. Kristally	M	151	w	0.19 ± 0.009	133.1 ± 4.8	3.38 ± 0.21	3.15 ± 0.23	70.42 ± 1.16	7.35 ± 0.62	0.62 ± 0.097	1.19 ± 0.17	5.33 ± 0.25	496.1 ± 12.3	267.9 ± 2.8	0.34 ± 0.070	0.56 ± 0.027	5.65 ± 0.34	1.78 ± 0.19	67.68 ± 1.11	244.9 ± 2.9	40.21 ± 0.43	0.57 ± 0.017	3.34 ± 0.23	0.22 ± 0.021	89.81	30.26 ± 1.16	0.61 ± 0.016	66.5 ± 1.6	1330 ± 23	2.36 ± 0.27	3.73 ± 0.24	3.17 ± 0.25	32 ± 11

**No$_{sample}$**	**Grape Type**	**Region of Poland**	**Location [Masl]**	**Color**	**Metal Content (µg/L) or (mg/L) /Isotope Mass (amu)**																													
					**[µg/L]**																								**[mg/L]**					
					**Ag 107**	**Al 27**	**As 75**	**Zr 90**	**Ba 138**	**Bi 209**	**Cd 111**	**Co 59**	**Cr 52**	**Cu 63**	**Fe 56**	**Hg 201**	**V 51**	**Li 7**	**Mo 98**	**Na 23**	**Ni 60**	**Pb 206**	**Sb 121**	**Se 78**	**Sn 115**	**Sr 88**	**Ti 47**	**Tl 205**	**Mg 25**	**K 41**	**Mn 55**	**B 10**	**Zn 64**	**Ca 40**
23 W	Seywal Blanc	M	151	w	0.16 ± 0.005	1589 ± 19.8	3.63 ± 0.23	3.34 ± 0.24	113.4 ± 2.4	9.01 ± 0.71	1.14 ± 0.16	1.99 ± 0.26	8.72 ± 0.34	83.71 ± 1.91	826.1 ± 23.8	0.34 ± 0.072	5.09 ± 0.31	8.09 ± 0.45	2.29 ± 0.21	463.1 ± 14.9	149.9 ± 2.4	17.28 ± 0.41	0.77 ± 0.018	4.47 ± 0.28	0.74 ± 0.034	211.1 ± 7.0	30.14 ± 1.11	0.56 ± 0.020	59.4 ± 1.5	1020 ± 22	1.47 ± 0.20	1.78 ± 0.15	3.45 ± 0.27	58.8 ± 1.4
24 W	St.Pepin. La Crescent	M	151	w	0.23 ± 0.011	91.61 ± 3.5	3.21 ± 0.19	1.08 ± 0.10	152.5 ± 2.7	7.17 ± 0.79	2.54 ± 0.32	1.17 ± 0.21	5.75 ± 0.29	400.2 ± 11.5	261.2 ± 3.1	0.35 ± 0.073	0.47 ± 0.23	0.53 ± 0.30	2.27 ± 0.20	5.33 ± 0.49	17.65 ± 1.19	6.77 ± 0.19	0.39 ± 0.012	4.35 ± 0.27	<0.021	196.1 ± 6.5	33.53 ± 1.14	0.72 ± 0.019	130 ± 2.5	1220 ± 27	3.94 ± 0.32	6.35 ± 0.39	4.96 ± 0.29	74.1 ± 1.8
1R	Allegro	W P	125	r	0.06 ± 0.003	415.1 ± 9.8	4.79 ± 0.23	1.22 ± 0.11	266.3 ± 4.9	2.51 ± 0.22	0.55 ± 0.091	1.81 ± 0.26	6.98 ± 0.32	47.03 ± 0.51	711.2 ± 21.8	0.34 ± 0.080	4.28 ± 0.26	15.37 ± 1.20	2.04 ± 0.22	3805 ± 45	35.17 ± 1.29	54.16 ± 0.43	0.34 ± 0.011	2.91 ± 0.19	0.136 ± 0.018	391.4 ± 5.2	32.71 ± 1.15	0.99 ± 0.019	82.9 ± 1.7	960 ± 14	2.78 ± 0.28	5.92 ± 0.25	1.66± 0.12	55.6 ± 1.5
2R	Regent. Rondo	W P	125	r	0.04 ± 0.001	276.1 ± 6.8	4.72 ± 0.23	1.09 ± 0.099	303.5 ± 5.2	2.39 ± 0.24	0.83 ± 0.14	1.91± 0.27	5.89 ± 0.31	10.51 ± 0.24	311.1 ± 2.9	0.34 ± 0.075	0.61 ± 0.023	6.48 ± 0.67	0.59 ± 0.034	3287 ± 42	29.11 ± 1.25	55.55 ± 0.43	0.26 ± 0.011	3.06 ± 0.21	<0.021	488.1 ± 6.2	35.41 ± 1.14	0.82 ± 0.017	108 ± 1.9	1190 ± 19	2.64 ± 0.26	4.90 ± 0.22	4.19 ± 0.30	50.4 ± 1.3
3R	Rondo	KP	74	r	3.85 ± 0.14	146.2 ± 7.8	3.62 ± 0.19	1.01± 0.11	95.41 ± 1.21	3.03 ± 0.23	0.42 ± 0.087	2.39 ± 0.32	7.15 ± 0.34	<0.268	290.4 ± 2.8	0.35 ± 0.069	0.03 ± 0.0021	9.33 ± 0.61	0.31 ± 0.021	311.1 ± 10.1	66.51 ± 1.51	12.12 ± 0.32	0.17 ± 0.011	3.75 ± 0.23	<0.021	212.1 ± 7.0	28.51 ± 1.00	0.76 ± 0.019	95.5 ± 1.7	1820 ± 34	1.56 ± 0.21	6.31 ± 0.38	4.87 ± 0.31	60 ± 1.6
4R	Regent	KP	74	r	0.03 ± 0.003	131.3 ± 4.8	3.47 ± 0.24	0.98 ± 0.034	79.31 ± 1.18	2.72 ± 0.20	0.39 ± 0.076	1.62 ± 0.25	6.54 ± 0.29	27.61 ± 0.34	268.1 ± 2.7	0.33 ± 0.068	<0.003	5.18 ± 0.59	0.58 ± 0.026	308.1 ± 10.8	49.31 ± 1.45	7.69 ± 0.19	0.14 ± 0.010	4.68 ± 0.32	<0.021	164.1 ± 6.5	28.32 ± 1.09	0.78 ± 0.020	98.9 ± 1.8	2050 ± 28	1.20 ± 0.16	5.98 ± 0.24	3.68 ± 0.27	56.6 ± 1.5

**No$_{sample}$**	**Grape Type**	**Region of Poland**	**Location [Masl]**	**Color**	**Metal Content (μg/L) or (mg/L) /Isotope Mass (amu)**																													
					**[μg/L]**																								**[mg/L]**					
					**Ag 107**	**Al 27**	**As 75**	**Zr 90**	**Ba 138**	**Bi 209**	**Cd 111**	**Co 59**	**Cr 52**	**Cu 63**	**Fe 56**	**Hg 201**	**V 51**	**Li 7**	**Mo 98**	**Na 23**	**Ni 60**	**Pb 206**	**Sb 121**	**Se 78**	**Sn 115**	**Sr 88**	**Ti 47**	**Tl 205**	**Mg 25**	**K 41**	**Mn 55**	**B 10**	**Zn 64**	**Ca 40**
5R	Regent	P	120	r	0.03 ± 0.002	201.1 ± 10.8	3.77 ± 0.23	1.39 ± 0.11	85.35 ± 1.20	3.63 ± 0.21	0.24 ± 0.056	0.87 ± 0.067	17.87 ± 0.75	<0.268	444.5 ± 12.3	0.33 ± 0.073	1.19 ± 0.11	2.31 ± 0.29	4.47 ± 0.28	219.1 ± 7.9	35.22 ± 1.23	11.61 ± 0.17	0.15 ± 0.011	2.58 ± 0.25	<0.021	225.5 ± 7.9	30.07 ± 1.12	0.51 ± 0.013	78.4 ± 1.6	1980 ± 19	0.405 ± 0.091	5.34 ± 0.27	6.27 ± 0.34	78.2 ± 1.8
6R	Pinot Noir.	KP	92	r	0.07 ± 0.004	292.1 ± 7.9	3.38 ± 0.22	1.25 ± 0.10	76.87 ± 1.12	3.03 ± 0.26	0.23 ± 0.051	0.45 ± 0.043	7.99 ± 0.34	<0.268	745.6 ± 23.8	0.33 ± 0.076	0.48 ± 0.12	34.01 ± 1.20	4.96 ± 0.429	460.4 ± 16.4	13.61 ± 1.16	9.61 ± 0.17	0.28 ± 0.013	12.85 ± 0.87	<0.021	297.1 ± 7.8	27.11 ± 1.07	0.63 ± 0.017	74 ± 1.5	1740 ± 30	1.51 ± 0.22	8.31 ± 0.39	3.63 ± 0.26	86.3 ± 1.9
7R	Rondo	SC	320	r	0.02 ± 0.001	462.1 ± 10.2	7.63 ± 0.43	4.02 ± 0.27	300.3 ± 5.1	2.19 ± 0.19	0.68 ± 0.097	3.25 ± 0.33	15.41 ± 0.70	77.82 ± 0.64	1232 ± 31	0.34 ± 0.071	5.09 ± 0.24	12.16 ± 0.67	3.26 ± 0.26	2393 ± 29	139.9 ± 2.4	13.02 ± 0.21	0.41 ± 0.015	3.74 ± 0.34	<0.021	1389 ± 12.2	30.91 ± 1.14	0.85 ± 0.019	106 ± 1.9	2020 ± 31	2.62 ± 0.27	11.8 ± 0.52	2.10 ± 0.21	81.6 ± 1.9
8R	Regent	SC	320	r	0.02 ± 0.002	680.7 ± 17.8	9.08 ± 0.37	2.16 ± 0.26	285.1 ± 4.9	1.84 ± 0.17	0.71 ± 0.099	2.78 ± 0.33	11.92 ± 0.49	15.63 ± 0.34	527.2 ± 13.0	0.33 ± 0.070	8.06 ± 0.29	18.91 ± 0.56	1.09 ± 0.099	2042 ± 30	132.5 ± 2.1	14.35 ± 0.22	0.29 ± 0.013	2.74 ± 0.23	<0.021	826.5 ± 8.9	28.55 ± 1.05	1.31 ± 0.13	106 ± 2.0	1670 ± 19	2.84 ± 0.28	12.1 ± 0.51	2.29 ± 0.19	66.4 ± 1.6
9R	Marechal Foch, Leon Millot	M	224	r	0.02 ± 0.001	345.5 ± 9.6	3.83 ± 0.17	1.77 ± 0.18	151.1 ± 3.2	1.27 ± 0.12	0.67 ± 0.095	2.52 ± 0.30	6.36 ± 0.28	<0.268	1064 ± 30	0.32 ± 0.068	2.89 ± 0.21	2.97 ± 0.29	0.98 ± 0.078	323.1 ± 10.2	81.61 ± 1.65	16.54 ± 0.20	0.14 ± 0.011	3.42 ± 0.30	<0.021	526.5 ± 6.2	28.16 ± 1.05	1.29 ± 0.18	106 ± 1.9	2050 ± 28	0.952 ± 0.20	11.9 ± 0.49	5.57 ± 0.30	78.3 ± 1.8
10 R	Frontenac	M	151	r	0.05 ± 0.003	806.6 ± 22.8	7.45 ± 0.25	2.55 ± 0.21	514.1 ± 12.3	4.04 ± 0.21	1.22 ± 0.18	2.42 ± 0.29	6.57 ± 0.26	<0.268	1002 ± 29	0.33 ± 0.069	19.21 ± 0.45	1.61 ± 0.20	3.71 ± 0.37	279.1 ± 9.8	63.42 ± 1.49	16.97 ± 0.19	0.48 ± 0.014	4.57 ± 0.34	<0.021	405.8 ± 6.2	28.51 ± 1.11	0.58 ± 0.023	91.4 ± 1.8	1630 ± 21	4.560 ± 0.35	4.49 ± 0.21	2.21 ± 0.17	105 ± 2.5

**No$_{sample}$**	**Grape Type**	**Region of Poland**	**Location [Masl]**	**Color**	**Metal Content (µg/L) or (mg/L) /Isotope Mass (amu)**																													
					**[µg/L]**																								**[mg/L]**					
					**Ag 107**	**Al 27**	**As 75**	**Zr 90**	**Ba 138**	**Bi 209**	**Cd 111**	**Co 59**	**Cr 52**	**Cu 63**	**Fe 56**	**Hg 201**	**V 51**	**Li 7**	**Mo 98**	**Na 23**	**Ni 60**	**Pb 206**	**Sb 121**	**Se 78**	**Sn 115**	**Sr 88**	**Ti 47**	**Tl 205**	**Mg 25**	**K 41**	**Mn 55**	**B 10**	**Zn 64**	**Ca 40**
11 R	Frontenac	M	151	r	0.04 ± 0.001	217.7 ± 4.8	6.41 ± 0.27	0.99 ± 0.056	373.1 ± 9.3	3.11 ± 0.20	0.73 ± 0.097	0.42 ± 0.054	4.62 ± 0.22	404.1 ± 11.9	715.1 ± 23.1	0.34 ± 0.074	0.11 ± 0.01	0.57 ± 0.11	3.25 ± 0.30	16.02 ± 1.11	33.31 ± 1.21	79.16 ± 0.79	0.32 ± 0.013	2.83 ± 0.28	<0.021	420.8 ± 6.1	27.32 ± 1.09	0.62 ± 0.021	99.2 ± 1.7	2100 ± 34	3.81 ± 0.31	6.03 ± 0.27	4.52 ± 0.27	96.3 ± 2.4
12 R	Regent	M	151	r	0.02 ± 0.0005	110.4 ± 3.1	1.93±0.12	1.08 ± 0.097	206.2 ± 4.2	2.51 ± 0.10	0.42 ± 0.061	0.22 ± 0.021	3.74 ± 0.20	<0.268	584.7 ± 17.8	0.33 ± 0.059	<0.003	1.97 ± 0.18	0.31 ± 0.021	118.3 ± 2.8	18.46 ± 1.129	8.97 ± 0.49	0.11 ± 0.010	1.31 ± 0.17	<0.021	262.1 ± 7.9	25.13 ± 1.04	0.63±0.020	96.5±1.9	1500 ± 19	2.22 ± 0.29	2.54 ± 0.19	0.87 ± 0.034	53.9 ± 1.5
13 R	Regent. Frontenac	M	151	r	0.17 ± 0.005	341.2 ± 7.2	2.43 ± 0.14	1.03 ± 0.067	307.3 ± 5.2	2.03 ± 0.14	0.66 ± 0.091	1.58 ± 0.26	8.31 ± 0.40	91.91 ± 1.34	861.9 ± 23.8	0.33 ± 0.067	0.43 ± 0.013	2.18 ± 0.20	5.26 ± 0.40	152.4 ± 7.4	73.99 ± 1.51	7.62 ± 0.54	0.25 ± 0.012	2.12 ± 0.19	<0.021	265.1 ± 7.6	27.21 ± 1.02	0.62 ± 0.023	140 ± 2.5	2380 ± 40	4.62 ± 0.42	4.31 ± 0.25	0.29 ± 0.011	83.5 ± 1.8
14 R	Heridian	M	151	r	0.06 ± 0.0008	294.6 ± 5.8	4.49 ± 0.17	1.86 ± 0.11	746.3 ± 14.5	7.32 ± 0.71	0.62 ± 0.090	0.44 ± 0.056	10.81 ± 0.55	475.1 ± 12.5	752.8 ± 21.8	0.31 ± 0.059	0.25 ± 0.011	0.42 ± 0.056	4.64 ± 0.37	12.21 ± 1.09	61.24 ± 1.47	46.11 ± 0.43	0.83 ± 0.016	3.67 ± 0.26	0.81 ± 0.012	560.5 ± 8.2	26.24 ± 1.09	0.64 ± 0.019	139 ± 2.4	2240 ± 37	9.22 ± 0.56	7.20 ± 0.32	0.38 ± 0.009	98.2 ± 1.9
15 R	Leon Millot. Marechal Foch.	M	151	r	<0.001	530.8 ± 17.8	5.52 ± 0.25	2.12 ± 0.15	507.1 ± 13.2	1.07 ± 0.10	0.51 ± 0.091	1.53 ± 0.25	7.64 ± 0.38	41.81 ± 0.49	482.8 ± 11.8	0.31 ± 0.057	7.81±0.37	1.55 ± 0.21	1.74 ± 0.21	138.3 ± 7.1	42.51 ± 1.42	22.21 ± 0.36	0.24 ± 0.011	4.33 ± 0.34	<0.021	472.2 ± 7.4	24.55 ± 1.04	0.71 ± 0.023	98.2 ± 1.9	97.1 ± 1.9	3.94 ± 0.32	3.83 ± 0.23	0.121 ± 0.024	72.1 ± 1.7
16 R	Leon Millot.	M	151	r	<0.001	104.1 ± 4.3	3.54 ± 0.23	0.98 ± 0.045	593.1 ± 13.3	0.42 ± 0.011	0.44 ± 0.071	0.68 ± 0.067	3.56 ± 0.20	42.44 ± 0.50	782.5 ± 23.8	0.31 ± 0.064	<0.003	0.95 ± 0.040	0.86 ± 0.021	19.15 ± 1.00	32.82 ± 1.32	34.71 ± 0.21	0.12 ± 0.010	4.02 ± 0.36	<0.021	700.3 ± 8.2	28.35 ± 1.11	0.71 ± 0.024	106 ± 1.9	2840 ± 39	4.32 ± 0.35	7.05 ± 0.40	0.399 ± 0.034	93.2 ± 2.0

No$_{sample}$	Grape Type	Region of Poland	Location [Masl]	Color	Metal Content (μg/L) or (mg/L) /Isotope Mass (amu)																													
					[μg/L]																								[mg/L]					
					**Ag 107**	**Al 27**	**As 75**	**Zr 90**	**Ba 138**	**Bi 209**	**Cd 111**	**Co 59**	**Cr 52**	**Cu 63**	**Fe 56**	**Hg 201**	**V 51**	**Li 7**	**Mo 98**	**Na 23**	**Ni 60**	**Pb 206**	**Sb 121**	**Se 78**	**Sn 115**	**Sr 88**	**Ti 47**	**Tl 205**	**Mg 25**	**K 41**	**Mn 55**	**B 10**	**Zn 64**	**Ca 40**
17 R	Marechal Foch.	M	151	r	<0.001	336.1 ± 12.1	2.18 ± 0.19	1.15 ± 0.13	474.1 ± 12.5	1.35 ± 0.091	0.93 ± 0.16	1.83 ± 0.28	4.37 ± 0.22	35.47 ± 0.46	791.5 ± 25.9	0.32 ± 0.065	<0.003	4.12 ± 0.32	1.46 ± 0.20	90.12 ± 2.13	30.34 ± 1.26	8.79 ± 0.19	0.11 ± 0.010	1.51 ± 0.21	<0.021	522.1 ± 8.0	27.21 ± 1.11	0.72 ± 0.020	134 ±2.3	3170 ± 51	7.07 ± 0.45	5.16 ± 0.24	0.449 ± 0.31	86.6 ± 1.9
18 R	St.Croix. Sabrevois	M	151	r	<0.001	109.1 ± 2.2	2.91 ± 0.17	0.93 ± 0.089	478.1 ± 12.3	0.17 ± 0.010	0.32 ± 0.045	0.16 ± 0.019	4.01 ± 0.21	13.69 ± 0.84	249.1 ± 2.8	0.31 ± 0.060	<0.003	4.11 ± 0.40	0.41 ± 0.018	98.48 ± 2.43	23.55 ± 1.17	11.31 ± 0.17	0.16 ± 0.013	1.55 ± 0.12	<0.021	478.1 ± 6.2	25.72 ± 1.06	0.76 ± 0.022	115 ± 2.1	3250 ± 54	5.13 ± 0.43	6.60 ± 0.30	0.075 ± 0.005	77 ± 1.8
19 R	Rondo.Regent	KP	92	r	0.03 ± 0.001	602.1 ± 17.8	6.21 ± 0.42	1.11 ± 0.094	60.51 ± 1.10	0.66 ± 0.011	0.58 ± 0.091	0.66 ± 0.067	17.03 ± 0.75	1284 ± 17	891.2 ± 23.8	0.32 ± 0.058	2.12 ± 0.21	29.91 ± 1.0	2.49 ± 0.28	380.9 ± 10.9	64.17 ± 1.45	349.9 ± 9.12	1.47 ± 0.14	11.55 ± 0.87	<0.021	197.1 ± 7.9	24.71 ± 1.05	0.57 ± 0.019	66.6 ± 1.4	1330 ± 23	2.27 ± 0.27	6.03 ± 0.29	2.409 ± 0.21	68.9 ± 1.6
20 R	Rondo	SC	320	r	0.03 ± 0.003	2489 ± 35.8	7.57 ± 0.46	12.32 ± 0.56	200.6 ± 4.2	1.48 ± 0.10	0.45 ± 0.067	1.89 ±	13.12 ± 0.61	1101 ± 16	2209 ± 39	0.32 ± 0.072	6.03 ± 0.28	18.94 ± 0.98	1.96 ± 0.21	3554 ± 42	138.9 ± 2.1	23.35 ± 0.26	0.54 ± 0.015	1.68 ± 0.14	<0.021	610.1 ± 8.2	23.81 ±1.11	0.95 ± 0.024	68.5 ± 1.3	1750 ± 19	2.19 ± 0.28	8.73 ± 0.43	0.122 ± 0.001	88.1 ± 1.9

KP, Kuyavian-Pomerania; LP, Lesser Poland; M, Masovia; P, Pomerania; r, red; SC, Subcarpatia; w, white; WP, West Pomerania

Table S2. Calibration curve parameters, LOD and LOQ values of ICP-MS/ICP-OES analytical techniques.

Element	Mass	R	1st term	Const.	LOD [μg L^{-1}] 3s	LOQ [μg L^{-1}] 10s
Ag	107	0.99999	0.107421	-0.00542	0.001	0.003
Al	27	0.99997	0.037549	-1.30754	0.031	0.103
As	75	0.99999	2.699769	-0.01012	0.006	0.021
B	11	0.99946	0.236112	-2.76755	0.418	1.395
Ba	138	0.98317	0.423326	4.314401	0.001	0.005
Bi	209	0.99939	0.153274	-0.09111	0.008	0.029
Cd	114	0.99957	0.311808	0.011723	0.002	0.007
Co	59	0.99999	0.143494	-0.0302	0.002	0.006
Cr	52	0.99896	0.30841	0.131909	0.036	0.123
Cu	63	0.99993	0.161124	-3.20904	0.368	1.227
Fe	56	0.99828	0.231216	2.390056	0.354	1.183
Hg	201	0.99731	0.586196	0.022925	0.004	0.016
Li	7	0.99996	0.120225	-0.06093	0.001	0.003
Mn	55	0.99974	0.584364	-1.07639	0.011	0.034
Mo	95	0.98929	0.467504	-0.07762	0.003	0.012
Na	23	0.99735	0.002412	-13.8966	0.032	0.106
Ni	60	0.98474	0.489872	-0.93239	0.03	0.115
Pb	206	0.99691	0.472782	0.402906	0.004	0.013
Sb	121	0.99962	0.41178	-0.01728	0.002	0.006
Se	78	0.99998	46.57817	-0.06415	0.061	0.202
Sn	115	0.99646	0.970044	-0.06483	0.021	0.069
Sn	116	0.99999	0.763471	-0.11474	0.020	0.067
Sr	88	0.98165	0.643907	4.663796	0.003	0.011
Ti	47	0.99617	14.98718	2.094714	0.082	0.274
Tl	205	0.99997	0.124699	0.040403	0.0002	0.0006
V	51	0.99844	0.52999	-0.02537	0.003	0.012
Zn	66	0.98432	0.680617	5.323809	0.017	0.059
Zr	90	1	0.175333	0.06953	0.002	0.009
Element	Wavelength	r	c	d	LOD [ug L^{-1}] 3s	LOQ [ug L^{-1}] 10s
Ca (7 L min^{-1})	393.3	0.99936	0.2546	-2.661	0.333	1.110
Ca (10 L min^{-1})	393.3	0.99922	0.2271	-0.0513	0.375	1.250
Mg (7 L min^{-1})	279.5	0.99997	0.0404	-0.4139	0.070	0.233
Mg (10 L min^{-1})	279.5	0.99891	0.0493	-0.0047	0.042	0.140
K (7 L min^{-1})	766.4	0.99854	0.5249	0.0434	1.465	4.884
K (10 L min^{-1})	766.4	0.99811	0.3773	-2.8110	0.332	1.108

Table S3. Validation report of ICP-MS analytical technique.

No Gas

Inten. / Peak

Elem	Mass	Inten. Min (kcps)	Inten.(kcps)	Inten. Judge	RSD Max(%)	Meas. RSD (%)	RSD Judge
Be	9	50	90.1738	Good	3	2.4	Good
In	115	200	390.4566	Good	3	0.7	Good
Bi	209	150	385.1826	Good	3	1.2	Good

Mass axis

Elem	Mass	Max gap (u)	Meas. gap (u)	Meas. ga Judge	Resoluti R value(	Meas. resolution (u)	Resolution Judge
Be	9	0.10	0.05	Good	0.9	0.77	Good
In	115	0.10	0.06	Good	0.9	0.73	Good
Bi	209	0.10	0.08	Good	0.9	0.74	Good

Oxide / Divalent / BG

Item	Formula	Unit	Max	Meas.	Judge	Output intensity	Standard element int
Ce Oxid	156 / 14	%	5.00	1.27	Good	5.5901	439.6427
Ce Dival	70.0 / 14	%	5.00	1.23	Good	5.5486	452.5296
BG	5	kcps	0.10000	0.00548	Good		
BG	220	kcps	0.01000	0.00044	Good		

Use Gas

Inten. / Peak

Elem	Mass	Inten. Min (kcps)	Inten.(kcps)	Inten. Judge	RSD Max(%)	Meas. RSD (%)	RSD Judge
Co	59	30	80.8148	Good	3	1.0	Good

Mass axis

Elem	Mass	Max gap (u)	Meas. gap (u)	Meas. ga Judge	Resoluti R value(	Meas. resolution (u)	Resolution Judge
Co	59	0.10	0.06	Good	0.9	0.73	Good

Oxide / Divalent / BG

Item	Formula	Unit	Max	Meas.	Judge	Output intensity	Standard element int
BG	78	kcps	0.10000	0.00448	Good		
